# Ventilator associated complications: observing implications of a new surveillance paradigm

**DOI:** 10.1186/2197-425X-3-S1-A941

**Published:** 2015-10-01

**Authors:** M Yuan, M Aaland, N Parekh

**Affiliations:** Anaesthesia and Critical Care, Queen Elizabeth Hospital Birmingham, Birmingham, United Kingdom

## Introduction

Surveillance for Ventilator Associated Pneumonia (VAP) is problematic. The CDC published a new surveillance framework [1] with two main goals.

• Broaden focus of surveillance beyond VAP to include other common ventilator-associated complications (VACs).

• Produce objective surveillance definitions using quantitative data based on changes in ventilator settings.

It introduces a hierarchy of surveillance targets:

1. Ventilator associated complications (VAC). Includes both pulmonary and non pulmonary complications.

2. Infection related (IVAC) complications with an infective component.

## Objectives

To gain an impression of rates of ventilator acquired complications using the new CDC criteria and impact on antibiotic prescription.

## Methods

• Inclusion Criteria: All consecutive patients intubated for at least 48 hours

• Exclusion Criteria: All elective post-cardiac surgery

• Follow Up: Until extubation or death

• Three random period of data collection

• In the first round data on 40 patients were captured

• Four months later in a second round a total of 18 patients were recruited.

## Results

**Figure 1:**
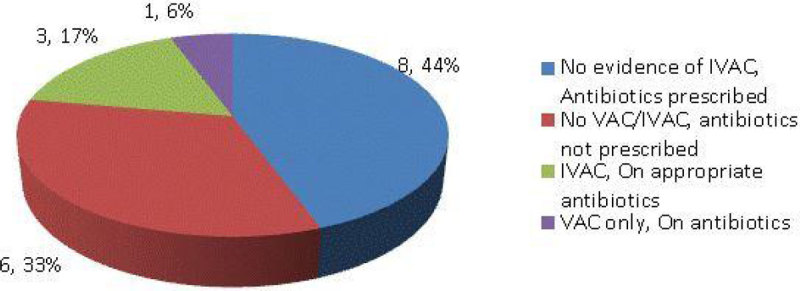
Figure 1

**Figure 2:**
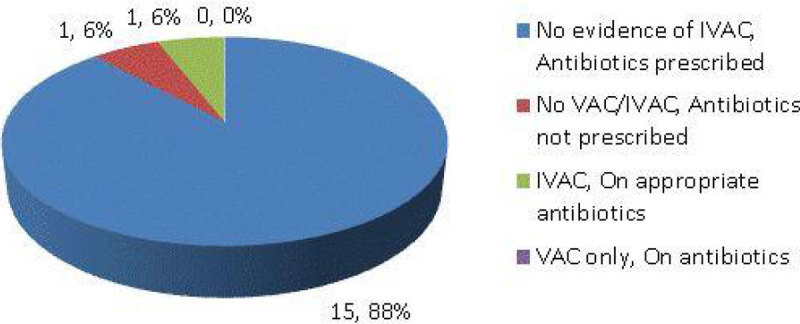
Figure 2

**Figure 3:**
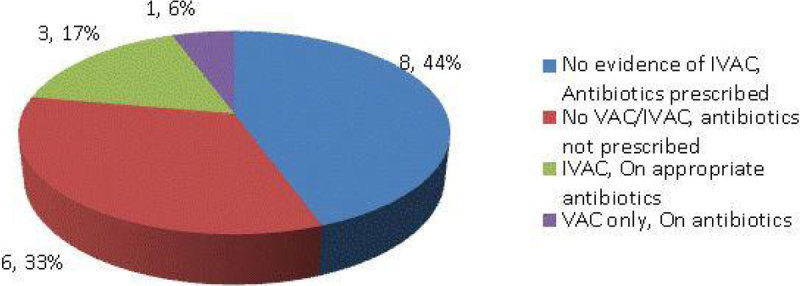
Figure 3

First round (Figure 1) on 23 patients shows VAC incidence of 7/23 (30.4%). In VAC group, 4 (17%) met IVAC criteria as possible pneumonia. Second round (Figure 2) enrolled 17 patients and shows VAC rate of 1/17 (11%) and that one case was possible pneumonia. Third round (Figure 3) enrolled 18 patients and shows a VAC rate of 4/18 (22%). In VAC group, 1 developed IVAC (6%) as possible pneumonia. Thus VAC rate varied from 11-30% but IVAC due to pneumonia ranged from 5-17%.

## Conclusions

• New CDC definition for VAC are easy to apply and removal of subjective criteria must be welcomed

• New definitions of IVAC allows clinicians to increase antibiotics free rate by 18-37% relatively

• In these 3 cohorts, several patients were treated with antibiotics despite no evidence to classify as IVAC. This can only be attributed to subjective decision and interpretation of chest x-ray

• The commonest indication for antibiotic prescription was non-pulmonary.

• Patients in possible pneumonia group on microbiology did not meet other IVAC criteria, highlighting issue of colonisation being treated with antibiotics

• IVAC metric thus has potential to identify outlier antibiotic prescribers

• Objective criteria to classify patients into VAC and IVAC has potential for automation in order to monitor the incidence of VAC, adding value to clinical dash board
